# Annotations

**Published:** 1899-11-11

**Authors:** 


### Annotations.

### A Consultative Institute.

A few months ago[1] we commented upon an attempt which was then being made to establish in Birmingham a consultative institute, which was to provide "consultations" at half a guinea for Birmingham working men. It was thought that the expenditure of the proposed institute would for the first year be about £1,500, and that there would be a sufficient number of consultations to yield an annual income of £3,000, and thus "leave, after payment of all expenses, a considerable profit." We had hoped that this ill-advised scheme had fallen through, but now we find that the proposal has come again to the front, and is this time taking a definite form. It is well, then, that we should insist how absolutely opposed any such plan is to the generally accepted rules as to professional remuneration. It is not in this case because the fees offered are low that we object to the scheme. Any medical man may accept what fees he likes—he may even take none at all, and yet he may not lose caste. It is a very different matter, however, for a medical man to allow some company, corporation, or other body to sell his services, and for its own purposes make what it can of them. This is the head and front of the offending of the Medical Aid Association, and of those who take service under them, and it is against this that the strongest protests have been and always will be made by all medical men who have the interests of their profession at heart. The Lord Mayor of Birmingham has been assured that, with the exception of a few of the senior and most important consultants, all the local consultants in Birmingham are prepared to see genuine working men for half a guinea. But that is a very different thing from farming out their services for a fixed salary to a lay body in order that the latter may make out of them "a considerable profit."

[1] The Hospital, May 20.

### Plague.

A cry is being raised in America that the British Empire is responsible for the plague, and that civilised nations must "insist" upon steps being taken by us to check the spread of the disease. The New York *Medical News*, writing on this subject, says: "The problem must not be dealt with merely on the lines of a civil misfortune to the Indian dependency, but England must realise the momentous interest which the disease has for the whole human race, and her plans for its complete eradication must be on a corresponding scale"; and further, "The civilised world has a right to demand that the British authorities in India shall omit nothing that can help to lessen the continual danger from the plague, which has now become a continual harassing threat." Now, it is very complimentary to England to look upon her as not only bound to act as the police of the ocean, but as the sanitary inspector and general mud-scraper for the world at large; but when we are told that "the British Empire has already been arraigned for its lack of vigilance and activity in failing to stamp out the disease at its inception" we would point out that the place of inception of plague, its permanent endemic home, does not lie in British territory at all, and that all this casting of blame upon England, which will no doubt be done by many other nations when once they begin to feel the touch of the scourge, is beside the mark. It is a pity, however, that in America, so advanced a country in many respects, the cry should still be for the old exploded method of quarantine as a means of stopping plague. "The only sure safety," we are told, "lies in not coming within the range of the plague's infectious influence; in other words, in quarantine." That is where the rub comes between England and other countries. They cry aloud for quarantine directly an infectious disease comes within hail of them, whereas we deal with the matter in quite another way. The Americans have very justly, and very comfortably, regarded Europe as a sort of buffer standing between them and plague. But now that the disease has crossed the boundary, has broken out on the very western edge of the European continent, and, finally, as is now reported, has jumped the Atlantic and shown itself in Brazil, the danger of the situation is coming home to them. There can be but little doubt that they are right in dreading the "direful havoc" which would be wrought among the slum and tenement-house population of their great cities, and among the negroes of the South, if once this insidious disease should gain a footing. But whether they are right in pinning their faith on quarantine, and in blaming other countries for letting the plague loose upon them, is another matter. With the rapid travel and the enormous trade of modern times quarantine is out of date.

### Subsidised Hospitals.

It is somewhat difficult for us in this country, where hospitals are either exclusively voluntary or exclusively rate-supported, to realise the complications which are introduced into the hospital problem in some countries, where not only do the hospitals receive a subsidy from the State or the municipality, but where at the same time patients who can be classified as "indigent" are paid for by the State. The constant abuses arising from the system of appropriating public funds for the support of private hospitals in various American cities are a frequent theme for writers in the American medical journals, but even they hardly afford such an example of the tendency to squeeze every possible penny out of the public funds which such systems encourage, as is afforded by the following incident which is related in a letter by Dr. Herbert Horrocks to the *Australasian Medical Gazette*: "A comparatively well-to-do man is admitted as an in-patient, and remains in the hospital for four weeks. He is stated by them to be indigent, and therefore the Government pays at the rate of £2 10s. a week for him. On leaving the hospital he makes a donation of £16 to the funds of the hospital, and they receive a subsidy of the same amount from the State. Result: £42 to the good." It should be mentioned that the State meets all donations by a grant of an equal amount, hence the temptation to set on foot the little jerrymandering scheme above described. If the man had openly paid his £4 a week as a pay patient, the hospital would have received nothing from the Government; but by taking him in as an "indigent," they not only get his keep of £2 10s. a week from the Government and his own payment of £4 a week (although in the form of a donation) from the man, but they get the latter met by a grant of an equal sum from the Government; an arrangement as ingenious as it is immoral.

